# Hemoptysis due to fungus ball after tuberculosis: A series of 21 cases treated with hemostatic radiotherapy

**DOI:** 10.1186/s12879-015-1288-y

**Published:** 2015-11-26

**Authors:** Lucas G Sapienza, Maria José L Gomes, Carmelindo Maliska, Antonio N Norberg

**Affiliations:** Radiation Oncology Department, Clínicas Oncológicas Integradas (COI-RJ), Rio de Janeiro, Brazil; Radiation Oncology Department, A.C. Camargo Cancer Center, São Paulo, Brazil; Radiation Oncology Department, Hospital Federal Servidores do Estado do Rio de Janeiro (HFSE-RJ), Rio de Janeiro, Brazil; Nuclear Medicine Department, Universidade Federal do Rio de Janeiro (UFRJ), Rio de Janeiro, Brazil; Infectology Department, Fundação Técnico-Educacional Souza Marques, Rio de Janeiro, Brazil

## Abstract

**Background:**

In patients who are not amenable to surgical resection (cavernostomy), it is difficult to achieve palliation of hemoptysis from pulmonary aspergilloma. There are only 9 cases with a short follow-up that have reported the use of radiotherapy for hemoptysis in this scenario.

**Methods:**

A retrospective series of 21 patients with chronic necrotizing pulmonary aspergillosis were treated with radiotherapy (20 Gray) from 1990 to 2002. The outcome measures were the period from tuberculosis treatment to the onset of hemoptysis, hemoptysis resolution rate, change in Zubrod performance status after 30 days of the completion of radiotherapy, local failure-free survival, and overall survival.

**Results:**

The median time between tuberculosis treatment and the onset of hemoptysis due to aspergilloma was 9 years. After radiotherapy, general status improved and the hemoptysis resolved in all patients. During the follow-up period, 4 failures occurred, with a 5-year local failure-free survival rate of 82 % and a 5-year overall survival rate of 59 %. Of these failures, 2 patients died due to recurrence of the hemoptysis, and 2 were rescued (using cavernostomy and reirradiation). The presence of chronic obstructive pulmonary disease (COPD) (*p = 0.021*) and female gender (*p = 0.032*) were negatively associated with overall survival. None of the variables was related to local control.

**Conclusions:**

Based on these long-term data, radiotherapy is a potential option for controlling bleeding due to fungus balls. Female patients and COPD were associated with lower survival.

## Background

Chronic pulmonary aspergillosis is a spectrum of diseases that are often difficult to differentiate, comprising from simple aspergilloma to lethal chronic necrotizing pulmonary aspergillosis (CNPA) [1–3]. CNPA is associated with a history of tuberculosis [4, 5], immunodeficiency during cancer treatment [6], chronic use of corticosteroids [7], and acquired immunodeficiency syndrome [8].

Treatment of CNPA is based on antifungal therapy [9, 10], but a subset of symptomatic patients benefit from surgical resection to control the disease and prevent and alleviate hemoptysis [11–14]. In cases of massive hemoptysis or poor surgical performance, therapeutic options are limited [15–18], and the prognosis is poor [5]. Arterial bronchial embolization is considered the standard of care for debilitated patients, with long-term findings published [16, 19], but an interventional radiologist is not available at all medical centers.

In this study, we present the long-term results of a series of 21 patients with low performance status who received radiation therapy alone to control their hemoptysis but continued to experience controlled disease.

## Methods

**Fig. 1:**
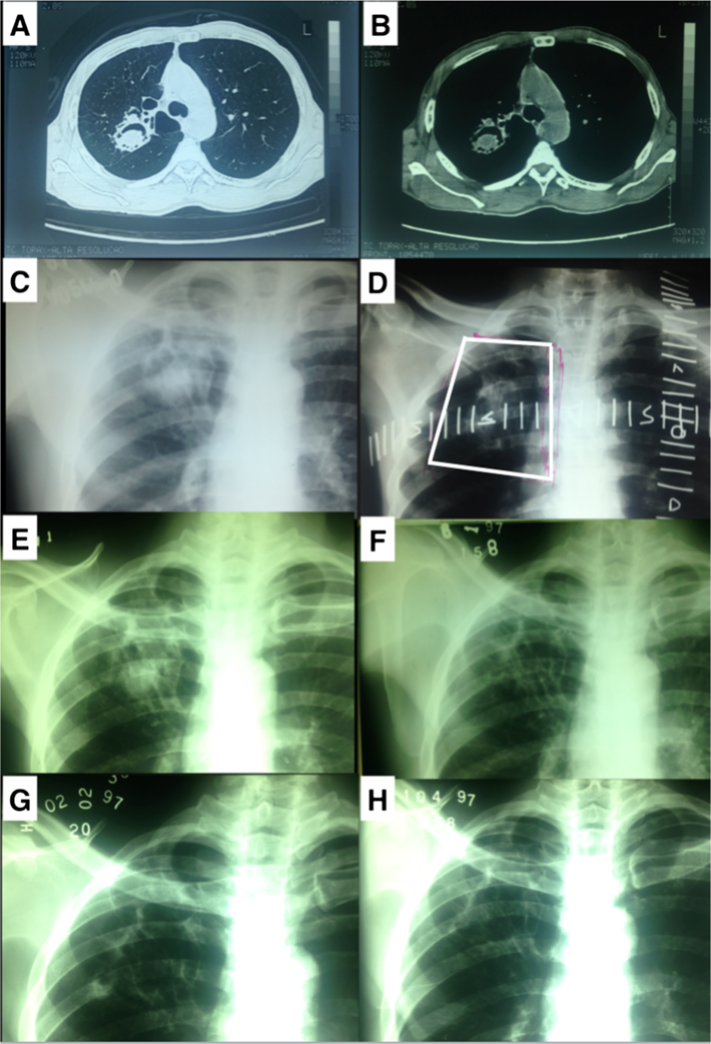
**a** 46-year-old man with a diagnosis of right superior lobe aspergilloma treated in August 1997. **a**) and **b**) Diagnostic computed tomography. **c**) Diagnostic x-ray. **d**) Localization x-ray. **e**) One-month follow-up x-ray. **f**) Five-month follow-up x-ray. **g**) Six-month follow-up x-ray. **h**) Eight-month follow-up x-ray

This retrospective study included 21 patients with CNPA who were treated from August 1990 to November 2002. At our department, the following triad is considered in making a diagnosis of CNPA: radiological image that is compatible with disease (cavitations in the lung apex with or without the presence of a fungus ball; Fig. 1); clinical condition (at least 2 of the following findings: hemoptysis, weight loss, cough, and anemia); and etiology (negative sputum acid-fast bacilli smear that is associated with a positive sputum culture for *Aspergillus fumigatus* or serology for detecting anti-*A. fumigatus* by double immunodiffusion) [20]. Although only 2 parameters are necessary to diagnose CNPA, the entire study group had all 3 criteria.

All cases except for one, who refused surgery, were considered to be inoperable after a multidisciplinary discussion between the chest surgery and radiation oncology departments and required local treatment due to hemoptysis. Per our institutional policy, we began to use irradiation to palliate hemoptysis in 1990, based on previous experience (see Discussion), and this approach was considered to be the standard of care at our hospital. The patients signed informed consent forms for treatment, per our institution’s guidelines. The ethics research committee of Hospital Federal Servidores do Estado do Rio de Janeiro (HFSE), Rio de Janeiro, Brazil, approved this study.

Radiation therapy was administered with a Cobalt-60 gamma unit (Gammatron S 80, Siemens, Munich, Germany). The volume of treatment consisted of the lesion, visualized using a simple x-ray or CT scan. A 1.0–2.0-cm margin was used to account for respiratory movements and positioning errors (Fig. 1). The prescribed dose was delivered to the isocenter of the lesion, 80 cm from the source, using 2 parallel opposed fields. The dose was 20 Gy, split into daily doses of 2 Gy, 5 days per week. In cases of persistent disease (radiological and serological or culture), an dose of 14 Gy was given. Recurrent cases were treated with a new course of 20 Gy, using the same dose fractionation.

Aspergillosis-free survival was analyzed after the first diagnosis of tuberculosis, based on the patients’ medical records and defined as the time between treatment for previous tuberculosis and the presentation of hemoptysis due to aspergilloma. Two patients were not included in this analysis, because they had no detailed medical records regarding the tuberculosis treatment.

Performance status was assessed at diagnosis and 30 days after the end of the treatment using the Zubrod score [21]. Patients were classified as: Zubrod 0 (asymptomatic), Zubrod 1 (ambulatory, restricted strenuous physical activity), Zubrod 2 (ambulatory, spending less than 50 % of the time in bed), Zubrod 3 (more than 50 % of the time in bed), and Zubrod 4 (fully bed-bound).

We assessed the following outcomes: change in performance status after 30 days of treatment, local failure-free survival, and overall survival. Local failure was defined as the presence of hemoptysis, associated with maintenance of positive serology or culture of *A. fumigatus*. Due to the limitations of a retrospective analysis of medical records, such information as the volume of bleeding, pulmonary function, and diagnostic criteria for COPD were unavailable.

We also determine the influence of the following variables, based on fungus ball relapse and overall survival: age (under vs over 45 years), gender (male *vs* female), COPD (present *vs* absent), laterality (unilateral *vs* bilateral), pretreatment Zubrod performance status (ZPS) (≤ 2 *vs* > 2), and post-treatment ZPS (0 *vs* ≥ 1). The data were presented in Kaplan-Meier plots, and factor analysis was performed by Cox regression for all of the variables above in the univariate model. Only variables with *p < 0.06* were tested in the multivariate model. The difference in performance status between groups was analyzed by McNemar’s test for cases with ZPS ≥ 3.

## Results

After the episode of massive hemoptysis, the mean and median follow-up of all patients was 73.5 months and 25 months, respectively (range: 1.3-281 months). Other patient characteristics are listed in Table 1.

**Table 1:** Patients characteristics

Age	Media 47 years (24–79)
Sex	
Male	11 (52 %)
Female	10 (48 %)
History of Tuberculosis (years ago)	
0-9 y	9 (47 %)
10-15 y	6 (32 %)
>15 y	4 (21 %)
COPD	
Yes	4 (19 %)
No	17 (81 %)
Disease localization	
Right	8 (38 %)
Left	8 (38 %)
Bilateral	5 (24 %)
Symptons	
Hemoptysis	21 (100 %)
Cough	19 (90 %)
Weight loss	18 (86 %)
Anemia	18 (86 %)
Zubrod performance status (initial)	
1	2 (9.5 %)
2	11 (52.4 %)
3	4 (19.05 %)
4	4 (19.05 %)

### Tuberculosis history

**Fig. 2:**
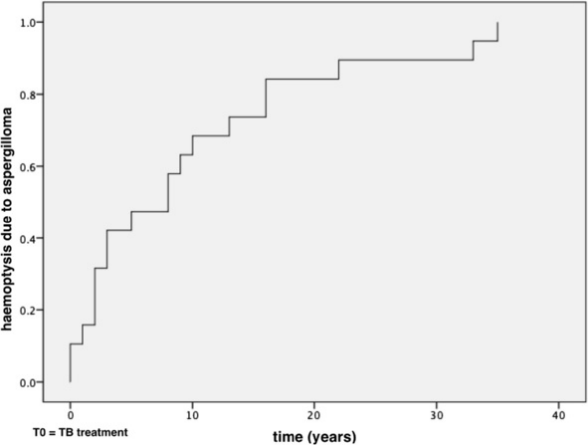
Time to diagnosis of aspergilloma after treatment for pulmonary tuberculosis. *n* = 19 cases

All patients had a history of treated pulmonary tuberculosis, and 6 patients had previously acquired tuberculosis more than once. The median time between the first diagnosis of tuberculosis (TB) and that of aspergilloma was 9 years, and the mean time was 11.74 years (Fig. 2). Gender was unrelated to differences in the incidence of fungus ball (*p = 0.23*).

### Symptomatic relief and toxicity

General pretreatment status improved in all cases 30 days after treatment. Eight patients (38 %) had an initial KPS ≥ 3, but no patient had KPS ≥ 3 after treatment (*p = 0.013*). All subjects experienced relief of the hemoptysis with 20-Gy treatment, except for 1 patient who required an additional dose of 14 Gy. There was no treatment-related toxicity, with the exception of mild cough.

### Local control

There were 4 local failures, which resulted in a 5-year local failure-free survival rate of 82 % in living patients. Of these failures, 2 patients died due to recurrence of hemoptysis, and 2 were rescued (1 by cavernostomy and other reirradiaed with 20 Gy). Fig. 1 (E,F,G,H) shows an example of complete radiological resolution of the lesion during the follow-up period. Our study did not identify cases with minor hemoptysis and negative serology. Patients without haemoptysis not perform serology tests.

### Overall survival

**Fig. 3:**
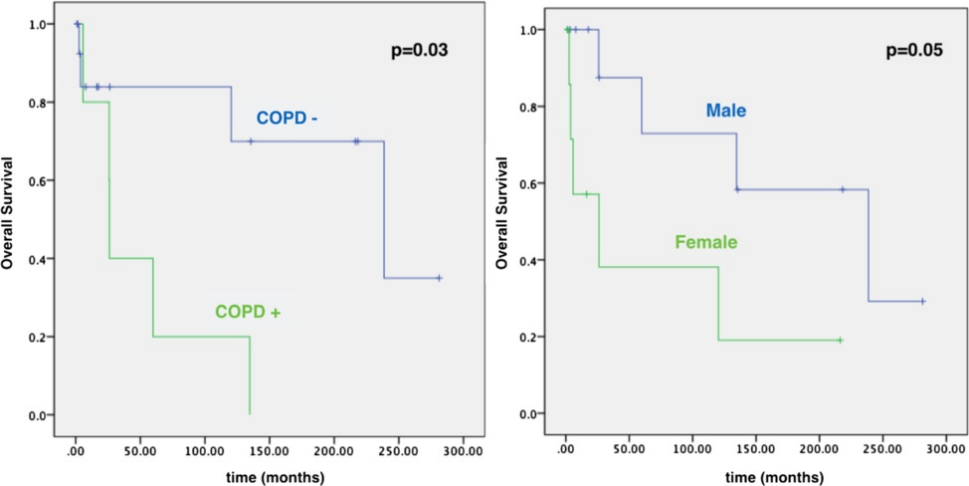
Kaplan-Meier curves demonstrating the negative impact of chronic pulmonary obstructive disease (COPD) and female gender on overall survival

Nine patients died during the follow-up, resulting in a 5-year overall survival rate of 59 %. The causes of death were relapse of aspergillosis in 2 cases, acute myocardial infarction in 1 case, COPD in 1 case, pneumonia in 1 case, and unspecified cause in 4 cases. Of the characteristics that we examined, the presence of COPD and female gender correlated negatively with overall survival (Fig. 3). No variables were related to local control (Table 2).

**Table 2:** Univariate and multivariate analysis

Overall Survival				
	Univariate		Multivariate	
Sex (male vs female)	*p* = 0.05	HR 0.24 (0.057 – 1.041)	*P* = 0.032	HR 0.176 (0.036 – 0.865)
COPD (no vs yes)	*p* = 0.03	HR 0.20 (0.048 – 0.896)	*P* = 0.021	HR 0.15 (0.03 – 0.756)
Uni x Bilateral	*p* = 0.26	HR 0.44 (0.105 – 1.873)	-	
Age (<45 y vs ≥45 y)	*p* = 0.77	HR 1.21 (0.325 – 4.533)	-	
ZPS pretreatment (0–2 vs 3/4)	*p* = 0.09	HR 0.30 (0.72 – 1.243)	-	
ZPS posttreatment (0–1 vs 2–4)	*p* = 0.07	HR 0.27 (0.63 – 1.164)	-	
Local Failure-Free Survival				
	Univariate		Multivariate	
Sex (male vs female)	*p* = 0.42	HR 0.44 (0.06 – 3.24)	-	
COPD (no vs yes)	*p* = 0.94	HR 1.079 (0.111 – 10.519)	-	
Uni x Bilateral	*p* = 0.15	HR 0.239 (0.033 – 1.718)	-	
Age (<45 y vs ≥45 y)	*p* = 0.29	HR 73.655 (0.023 - >100)	-	
ZPS pretreatment (0–2 vs 3/4)	*p* = 0.06	HR 0.114 (0.012 – 1.117)	-	
ZPS posttreatment (0–1 vs 2–4)	*p* = 0.33	HR 0.006 (<0.001 - >100)	-	

ZPS: Zubrod performance status

## Discussion

Radiation therapy was effective in treating this group of inoperable patients with CNPA, with benefits observed with regard to remission of hemoptysis symptoms, general condition of the patients, and disease control.

The colonization of cavities that originate from previous infection with pulmonary tuberculosis by the fungus *A. fumigatus* occurs in many patients and can lead to serious conditions, such as CNPA. According to data from 2012, pulmonary tuberculosis affects approximately 120,000 Brazilians of a population of approximately 200 million inhabitants. The prevalence of tuberculosis infection has declined gradually in recent decades [22]. However, in this study, 10 of 19 patients were diagnosed with aspergilloma over 10 years after the pulmonary tuberculosis infection, suggesting that the actual number of patients with pulmonary aspergilloma reflects the incidence of tuberculosis in the prior decade.

This study is the largest experience on the use of radiation therapy for fungus ball treatment, adding 21 subjects to the 9 cases that have been published. Initially, Shneerson et al. described the first case of massive hemoptysis in a patient with pulmonary aspergilloma, which was successfully treated with radiation therapy [23]. In 1994, a Spanish group reported another patient with a history of tuberculosis 2 years before the onset of hemoptysis, which was also treated successfully [24]. Twenty years after the first report, the use of radiation therapy for this condition was described in 5 new patients in South Africa [25]. In the United Kingdom, Glover et al. treated hemoptysis in a patient with aspergilloma due to p-ANCA-positive small-vessel vasculitis [26]. The first report in the United States was described in 2011 [27], regarding a patient with aspergilloma and chronic obstructive pulmonary disease—in whom the bleeding also resolved.

Based on these experiences, we recommend the use of 20 Gy in 10 daily applications, due to the low toxicity in our series and in the treatment of other chest conditions, such as initially favorable Hodgkin lymphoma [28]. Retreatment with 14 Gy in 7 applications was effective in the salvage of 1 patient who remained symptomatic with hemoptysis with positive Aspergillus serology postradiotherapy. Although we considered the possibility of in vivo fungicidal activity with the range of doses that we used (20–40 Gy), an in vitro study has suggested the need for doses that are above the therapeutic range to eliminate the infectious agent [29]. Further studies are needed to determine the radiation sensitivity of fungi, especially the genus *Aspergillus*, in vivo.

In addition to our study being retrospective and lacking a control group, other limitations include the use of cobalt devices that are not widely available and localization that was based on simple x-rays, which can generate failure of target coverage. Patients with a previous tuberculosis infection are common in developing countries, such as Brazil, but there might be differences compared with cases that are treated for cancer and rheumatic diseases in developed countries. This article did not report any autopsy findings in patients after death to determine the viability of *A. fumigatus* in the treated areas.

The optimal treatment for massive hemoptysis remains debated [30]. Bronchial artery embolization could be considered the standard approach, based on the increasing evidence of its efficacy [16, 19]. However, in cases for which an experienced interventional radiologist team is unavailble, hemostatic radiation therapy should be offered.

## Conclusions

Radiation therapy merits consideration as an reasonable, effective therapeutic option for controlling hemoptysis due to CNPA, with potentially positive effects on disease-free survival. Additional studies are needed to evaluate the efficacy of this treatment prospectively, after which radiotherapy can be compared with other treatment modalities, such as bronchial artery embolization.
